# Decline of Ambient Air Pollution Levels and Improved Respiratory Health in Swiss Children

**DOI:** 10.1289/ehp.8159

**Published:** 2005-06-21

**Authors:** Lucy Bayer-Oglesby, Leticia Grize, Markus Gassner, Kathy Takken-Sahli, Felix H. Sennhauser, Urs Neu, Christian Schindler, Charlotte Braun-Fahrländer

**Affiliations:** 1 Institute of Social and Preventive Medicine of the University of Basel, Basel, Switzerland; 2 School Health Service, Grabs, Switzerland; 3 School Health Service, Zürich, Switzerland; 4 University Children’s Hospital, Zürich, Switzerland; 5 Institute of Geography, University of Bern, Bern, Switzerland

**Keywords:** air pollution, children, cross-sectional surveys, decline, respiratory health, symptoms

## Abstract

The causality of observed associations between air pollution and respiratory health in children is still subject to debate. If reduced air pollution exposure resulted in improved respiratory health of children, this would argue in favor of a causal relation. We investigated whether a rather moderate decline of air pollution levels in the 1990s in Switzerland was associated with a reduction in respiratory symptoms and diseases in school children. In nine Swiss communities, 9,591 children participated in cross-sectional health assessments between 1992 and 2001. Their parents completed identical questionnaires on health status and covariates. We assigned to each child an estimate of regional particles with an aerodynamic diameter < 10 μg/m^3^ (PM_10_) and determined change in PM_10_ since the first survey. Adjusted for socioeconomic, health-related, and indoor factors, declining PM_10_ was associated in logistic regression models with declining prevalence of chronic cough [odds ratio (OR) per 10-μg/m^3^ decline = 0.65, 95% confidence interval (CI), 0.54–0.79], bronchitis (OR = 0.66; 95% CI, 0.55–0.80), common cold (OR = 0.78; 95% CI, 0.68–0.89), nocturnal dry cough (OR = 0.70; 95% CI, 0.60–0.83), and conjunctivitis symptoms (OR = 0.81; 95% CI, 0.70–0.95). Changes in prevalence of sneezing during pollen season, asthma, and hay fever were not associated with the PM_10_ reduction. Our findings show that the reduction of air pollution exposures contributes to improved respiratory health in children. No threshold of adverse effects of PM_10_ was apparent because we observed the beneficial effects for relatively small changes of rather moderate air pollution levels. Current air pollution levels in Switzerland still exceed limit values of the Swiss Clean Air Act; thus, children’s health can be improved further.

The causality of observed associations between air pollution and respiratory health in children is still subject to debate, although numerous studies have reported adverse effects of air pollution on the respiratory health of children, using indicators of general air pollution (Braun-Fahrländer et al. 1997; Chen et al. 1998; Gauderman et al. 2002; Horak et al. 2002; Hruba et al. 2001; McConnell et al. 1999) and of traffic-related air pollution (Brauer et al. 2002; Gehring et al. 2002; Hirsch et al. 1999; Janssen et al. 2003; Nicolai et al. 2003; van Vliet et al. 1997; Venn et al. 2001; Wjst et al. 1993). If it could be shown that reduced air pollution exposures improve the respiratory health of children, this would argue in favor of a causal relation. So far, only a few studies have investigated the expected beneficial effects of air pollution reduction on respiratory health in children. In cross-sectional analysis, the tremendous decline of coal combustion–related air pollution in East Germany after reunification was associated with a decline of respiratory symptoms (Heinrich 2003) and improved lung function (Frye et al. 2003) in children. In a cohort of children, those who moved within California to areas with lower PM_10_ (particles with an aerodynamic diameter < 10 μg/m^3^) levels showed increased lung function growth, whereas those moving to more polluted areas had a decreased growth (Avol et al. 2001). McConnell et al. (2003) observed that bronchitis symptoms, assessed yearly for 4 years in a cohort of children with asthma, varied with the yearly variability of PM_2.5_ (particles with an aerodynamic diameter < 2.5 μg/m^3^), nitrogen dioxide, and organic carbon.

In the first cross-sectional assessment of the Swiss Surveillance Program of Childhood Allergy and Respiratory Symptoms with Respect to Air Pollution and Climate (SCARPOL) in 1992–1993, Braun-Fahrländer et al. (1997) reported that rates of respiratory symptoms and diseases, adjusted for individual risk factors, were positively associated with PM_10_, NO_2_, and sulfur dioxide in children living in 10 urban, suburban, rural, and alpine areas of Switzerland. Since then, air pollution abatement measures (emission limits for industries, introduction of low-sulfur heating oil and catalytic converters) implemented after the Swiss Clean Air Act (1985) have led to declining air pollution levels in Switzerland [Swiss Agency for the Environment, Forest and Landscape (SAEFL) 2003; Kuebler et al. 2001]. In contrast to East Germany, where the tremendous air pollution decline in the 1990s went hand in hand with dramatic political and social changes, the political and social system in Switzerland has been very stable for many decades, which is an asset in our study. We hypothesize that if the health effects observed in SCARPOL in 1993 (Braun-Fahrländer et al. 1997) were causal, *a*) the observed reduction of PM_10_ in Switzerland since the first cross-section of SCARPOL would be associated with a reduction of prevalence rates of respiratory symptoms and diseases in the second health assessment phase, and *b*) the average reduction of symptom prevalence would be more pronounced in areas with stronger reduction of air pollution levels.

## Methods

### Study population and design.

In 10 Swiss communities covering a broad range of urbanization, air pollution levels, and climatic conditions, 10,397 school children (76.1%) ages 6–15 years have participated in cross-sectional, questionnaire-based health assessments between 1992 and 2001. For urban areas, we chose Lugano, Zürich, Bern, and Geneva; for suburban areas, Anières and Biel; for rural areas, Langnau, Payerne, and Rheintal; and for an alpine area, Montana. Because of the absence of PM_10_ data, we had to exclude children of Rheintal for this analysis, resulting in a sample of 9,591 children. The detailed recruiting procedure for the first cross-sectional health assessment in 1992–1993, which has also been applied for subsequent assessments, has been described previously (Braun-Fahrländer et al. 1997). Children of three school grades (first, fourth, and eighth) were recruited in the first phase in 1992–1993; in the second phase, one grade was enrolled each school year (first grade in 1998–1999, eighth grade in 1999–2000, and fourth grade in 2000–2001) (Table 1). This resulted in two repeated cross-sectional surveys for each age-group that are 6, 7, and 8 years apart for the first, eighth and fourth grade, respectively. The ethics committees of the Universities of Geneva and Bern approved the study protocol.

### Health assessment.

For all participating children, we collected identical parent-completed questionnaires on health status, family history of disease, spare-time activities, indoor exposures, and residential situation. The questionnaire included the core questions on asthma and allergy of the International Study of Asthma and Allergy in Childhood (ISAAC) (Asher et al. 1995). Definitions of symptoms and diseases examined in this analysis are given in Table 2.

### Assessment of air pollution exposures.

We assigned to each child an estimate of regional PM_10_ for the year preceding the questionnaire date, obtained from one fixed monitoring station in each community. Children were living within a few (3–5) kilometers of the monitors. Monitors were located in the centers of the communities, with the exception of the rural monitors in Payerne and Montana. Röösli et al. (2000, 2001) have demonstrated that in Switzerland, PM_10_ levels are homogeneously distributed within regions and are not significantly affected by local traffic, justifying the single-monitor approach for the assignment of PM_10_ exposures. Because PM_10_ measurements started in four communities not before 1993, we assigned annual means of 1993 to all children participating in the first cross-section (1992–1993 school year). Annual means of PM_10_ have been estimated for 1993 and for 1997–2000. We converted Harvard Impactor data of 1993 to DIGITEL HiVol values based on collocated measurements of the two monitors for 24 months (Krütli and Monn 1999). Between 1997 and 2000, PM_10_ was measured in the nine regions with DIGITEL HiVol samplers (DIGITEL 1999). In addition, we obtained temperature measurements from the fixed monitoring stations for calculating the number of cold days (days with the maximum temperature below zero degrees Celsius) for each region and year.

### Statistical analysis.

To analyze the association between change of air pollution levels and change of respiratory health, we used multivariate logistic regression models. For the children participating in the second health assessment phase (school years 1998–1999, and 2000–2001), change in PM_10_ was calculated as the difference between the assigned PM_10_ estimate and the 1993 baseline values corresponding to their area. For the children participating in the first health assessment phase (school year 1992–1993), change in PM_10_ was set to zero. In addition to change in PM_10_, a dummy variable for each region was included in the regression models. To test for community correlation possibly introduced by clustering of uncontrolled covariates, we also evaluated random-effect models.

For the nine health end points, we computed adjusted odds ratios (ORs) associated with a decline of 10 μg/m^3^ in PM_10_. *A priori*, our regression models also included those covariates that had an impact on the effect estimates or were identified as confounders of air pollution effects in the first cross-sectional analysis of 1992–1993 (Braun-Fahrländer et al. 1997). Covariates included

- Socioeconomic factors (age, sex, nationality, parental education, number of siblings, farming status)
- Health-related factors (low birth weight, breastfeeding, child who smokes, family history of asthma, bronchitis, and/or atopy)
- Indoor factors (mother who smokes, humidity, mode of heating and cooking, carpeting, pets allowed in bedroom)
- Avoidance behavior with respect to allergies (carpet or pets removed for health reasons)
- Questionnaire-related factors (person who completed questionnaire).

These covariates proved relevant in the multivariate model also for analyzing the impact of change of PM_10_ on respiratory symptoms. Age was included as a categoric variable (three groups according to school grades) because preliminary analysis suggested a nonlinear association between age and the evaluated health outcomes. In the first cross-section, all questionnaires were completed during wintertime to avoid confounding by season. The cross-sectional assessments of the second phase had to be spread over the whole school year for logistic reasons. A dummy variable for the month when the questionnaire was completed was included in the multivariate logistic regression models to adjust for possible reporting bias by season of the interview.

We evaluated whether secular trends had occurred between 1992–1993 and 1998–2001 that could be related to changing prevalence of the investigated symptoms and diseases—namely, climatic factors (milder or colder winters), participation rates, and mother’s concern about an association between environmental exposure and children’s respiratory health.

We further tested the final models for interactions between change of PM_10_ on the one hand and covariates such as age group, sex, family history of allergic diseases (asthma and/or atopy), asthma ever of child, smoker (child and/or mother), and indoor exposures (heating and/or cooking) on the other. The fit of the final models was evaluated.

To evaluate whether the average reduction of symptom prevalence is more pronounced in areas with stronger reduction of air pollution, we computed covariate-adjusted prevalence by community for the first (1992–1993) and second health assessment phase (1998–2001). To visualize the associations, we plotted the mean region-specific change in adjusted prevalence between the first and second phase against the respective mean change in PM_10_ levels. Corresponding Pearson correlation coefficients for the associations between these aggregate data were computed.

All analyses were conducted with Stata Statistical Software, Release 8.0.SE (StataCorp, College Station, TX, USA).

## Results

### PM_10_ levels, adjusted prevalence, and covariates 1992–2001.

**Figure 1:**
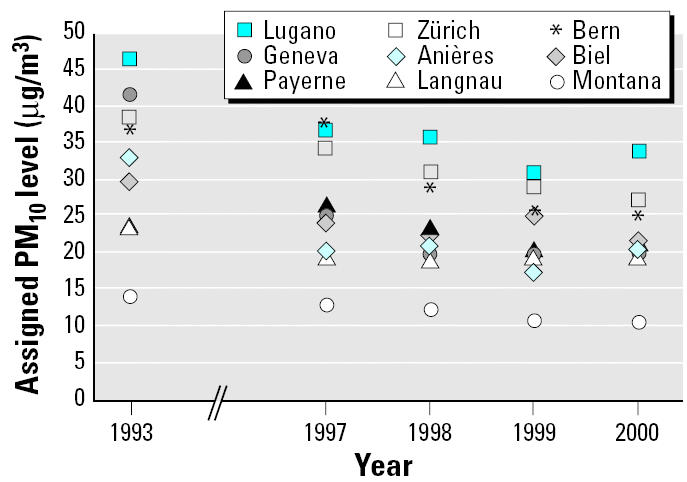
Annual means of PM_10_ levels*a* assigned to children of the first (1993) and second (1997–2000) health assessment phase in nine SCARPOL regions. ^a^Measured with DIGITEL HiVol Samplers. 1993 data converted from Harvard Impactor data.

Figure 1 shows PM_10_ levels at fixed monitoring sites in nine study regions of SCARPOL in 1993 and between 1997 and 2000. Across the nine study regions, the average decline of PM_10_ between 1993 and 2000 was 9.8 μg/m^3^ (29%). The average absolute decline in the urban and suburban areas Anières, Bern, Biel, Geneva, Lugano, and Zürich (12.7 μg/m^3^) was about three times as strong compared with the rural and alpine areas Langnau, Payerne, and Montana (4.0 μg/m^3^).

The adjusted prevalence of all investigated health end points declined between 1992–1993 and 1998–2001 (Table 3). Both the absolute and relative declines were stronger for the nonallergic outcomes chronic cough, bronchitis, common cold, nocturnal dry cough, and conjunctivitis symptoms (4.5–8.9% absolute decline of prevalence, on average, across the nine regions) compared with the allergy-associated end points sneezing during pollen season, asthma, and hay fever (0.4–1.7%). A tendency of a stronger absolute decline in suburban areas compared with rural/alpine areas was observed for the nonallergic, but not for the allergy-associated, outcomes.

Table 4 shows the distribution in the first (1992–1993) and second (1998–2001) health assessment phase of the covariates included in the multivariate models for analyzing the association between change of air pollution and change of prevalence. Excluded are children with missing data for one or more covariates. The most striking time trend is the increase in self-reported smoking of eighth graders from 6.4 to 16.3% (*p* < 0.0001). Mothers’ environmental concerns had declined on average from 78.9 to 75.6% (*p* = 0.001). The average annual number of cold days (days with the maximum temperature below zero degrees Celsius) had declined from 15 in 1992–1993 to 12 in 1998–2002 (*p* < 0.0001) across all study regions, with the strongest decline in Anières (from 10 to 3). An increase in the number of cold days was recorded in the alpine area Montana (from 21 to 38). Because the generally milder winters (with the exception of Montana) and the attenuated environmental concerns would be expected to move in the same direction as declining air pollution levels, that is, toward lower prevalence of reported symptoms and diseases, the logistic regression models were adjusted for the two secular trends. Participation rates in the four cross-sections (69.9, 82.4, 75.3, and 75.0%, respectively) indicated no secular trend.

### Change in PM_10_ exposure versus change in prevalence.

**Figure 2:**
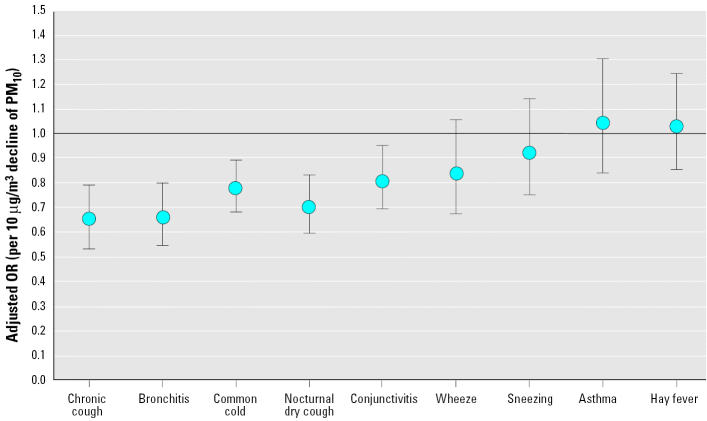
Adjusted ORs^a^ and 95% CIs of symptoms and respiratory diseases in SCARPOL associated with a decline of 10 μg/m^3^ PM_10_ levels. ^a^Adjusted for age, sex, nationality, parental education, number of siblings, farming status, low birth weight, breast-feeding, child who smokes, family history of asthma, bronchitis, and/or atopy, mother who smokes, indoor humidity, mode of heating and cooking, carpeting, pets allowed in bedroom, removal of carpet and/or pets for health reasons, person who completed questionnaire, month when questionnaire was completed, number of days with the maximum temperature < 0°C, belief of mother that there is an association between environmental exposures and children’s respiratory health, and region.

Figure 2 shows that declining levels of PM_10_ were associated with declining prevalence of chronic cough, bronchitis, common cold, nocturnal dry cough, and conjunctivitis symptoms. For wheezing, sneezing, asthma, and hay fever, no significant association could be seen with declining PM_10_ levels. We found no effect modification by age group, sex, family history of allergic diseases, asthma of child, smoking, or indoor exposures. Random effect models did not change the effect estimates.

Mothers’ concerns regarding air pollution and children’s respiratory health were significant predictors for reported bronchitis, common cold, nocturnal dry cough, conjunctivitis symptoms, wheeze, and asthma, whereas the number of cold days was not significantly associated with reported symptoms and diseases (data not shown). Without adjustment for the temporal trends of mothers’ beliefs (on individual level) and number of cold days (on area level), the effect estimates were slightly stronger for chronic cough, common cold, nocturnal dry cough, and conjunctivitis symptoms and reached significance for wheeze (data not shown). Besides change in PM_10_, the covariates age, family history of bronchitis, child’s smoking, indoor humidity, and removal of carpets were the strongest significant predictors for chronic cough and bronchitis, while for asthma and hay fever, this applied to sex, age, family history of asthma and atopy, and removal of carpets and pets (data not shown). Crude estimates were quite similar to adjusted ORs (data not shown). The fit of the models was generally satisfactory according to Hosmer-Lemeshow chi-square (8 d.f.).

**Figure 3:**
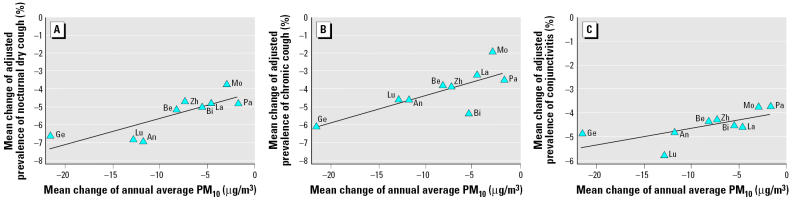
Mean change in adjusted prevalence^a^ (1998–2001 to 1992–1993) versus mean change in regional annual averages of PM_10_ (1997–2000 to 1993) for nocturnal dry cough, chronic cough, and conjunctivitis symptoms across nine SCARPOL regions. Abbreviations: An, Anières; Be, Bern; Bi, Biel; Ge, Geneva; La, Langnau; Lu, Lugano; Mo, Montana; Pa, Payerne; Zh, Zürich. ^a^Adjusted for age, sex, nationality, parental education, number of siblings, farming status, low birth weight, breastfeeding, child who smokes, family history of asthma, bronchitis, and/or atopy, mother who smokes, indoor humidity, mode of heating and cooking, carpeting, pets allowed in bedroom, removal of carpet and/or pets for health reasons, person who completed questionnaire, month when questionnaire was completed, number of days with the maximum temperature < 0°C, and belief of mother that there is an association between environmental exposures and children’s respiratory health.

Figure 3 illustrates that, on an aggregate level, across regions the mean change in adjusted prevalence of nocturnal dry cough is associated with the mean change in PM_10_ levels (*r*_Pearson_ = 0.81, *p* = 0.008). The strongest decline of adjusted prevalence of nocturnal dry cough was observed in Geneva, Lugano, and Anières, where the strongest reduction of PM_10_ had also been achieved. Similar associations were observed for chronic cough (*r* = 0.78; *p* = 0.02) and conjunctivitis symptoms (*r* = 0.69; *p* = 0.04) (Figure 3), whereas for common cold (*r* = 0.48; *p* = 0.19) and bronchitis (*r* = 0.10; *p* = 0.80), the associations across regions were weaker and not significant.

## Discussion

We showed that decreasing levels of PM_10_ were associated with declining prevalence rates of those respiratory symptoms and diseases associated with air pollution in the first cross-sectional analysis of SCARPOL (Braun-Fahrländer et al. 1997). The reduction in prevalence rates was larger in areas with a stronger decrease in PM_10_ levels. Decreasing environmental concerns of mothers (Swiss Society for Applied Social Research 2003) over time contributed to the observed decrease in respiratory symptoms and diseases but did not explain the association with air pollution. Adverse effects of PM_10_ have no apparent threshold, as we observed the beneficial effects for relatively small changes in rather moderate air pollution levels. We therefore conclude that even relatively small reductions in air pollution levels may improve children’s respiratory health.

Our findings are consistent with the improvement of nonallergic respiratory morbidity in children along with declining air pollution levels reported for East Germany (Heinrich et al. 2002; Kramer et al. 1999), although baseline levels and decline in Switzerland (SAEFL 2003) were much smaller. They are also in line with the few intervention studies that have investigated the impact of changing air pollution levels on children’s lung function growth (Avol et al. 2001; Frye et al. 2003; Neuberger et al. 2002) and bronchial responsiveness (Wong et al. 1998) and on mortality in adults (Clancy et al. 2002; Hedley et al. 2002). All these studies have found improved respiratory health or reduced respiratory and cardiovascular mortality after mitigation of ambient air pollution exposures. The consistency of these findings suggests that the observed associations between air pollution and respiratory health outcomes may be causal.

In our study, declining PM_10_ levels were not associated with changes in prevalence of asthma, hay fever, and sneezing during pollen season. No adverse effects of PM_10_ were observed for these allergy-associated health outcomes in cross-sectional analyses of SCARPOL (Braun-Fahrländer et al. 1997), and they have shown only a very small average decline in our study population and stable prevalence over the last decade in Swiss adolescents (Braun-Fahrländer et al. 2004). A similar contrast between nonallergic and allergy-associated health outcomes in children and declining air pollution levels has been reported by Kramer et al. (1999). Hirsch et al. (1999) reported significant associations of NO_2_, carbon monoxide, and benzene with bronchitis and morning cough but not with allergy-associated end points. A few studies using traffic counts or proximity to street as exposure proxy found positive associations with sensitization and allergy-related symptoms (Nicolai et al. 2003; van Vliet et al. 1997; Venn et al. 2001; Wjst et al. 1993). We cannot exclude such effects, but for our analysis we had no such data available.

Adjustment for the observed time trends of declining environmental concerns of mothers and reduced number of cold days over the study period did not markedly change the effect estimates. The monitoring of influenza epidemics by the Swiss Federal Office of Public Health (SFOPH) does not suggest a decrease in influenza between 1992 and 2001, which might have confounded our findings, but indicates random fluctuations between years (SFOPH 2001). The same is true for the number of hourly ozone concentrations exceeding 120 μg/m^3^ [Federal Commission for Air Hygiene (EKL) 2004]. For evaluation of the impact of other possible secular trends such as changes in health habits or medication use, we had no data available. Confounding of our cross-sectional findings by political or social time trends is very unlikely. In Switzerland, the system has been very stable throughout the study period (and was for many decades before), in contrast to the social changes that went hand in hand with air pollution reduction in East Germany (Frye et al. 2003; Heinrich 2003). Thus, uncontrolled confounding or secular trends are unlikely to explain our finings.

Our study is limited in that the comparison for each school grade is based on two points in time only, which are 6, 7, and 8 years apart for the first, eighth, and fourth graders, respectively. The difference in absolute change between the three age groups has been taken into account by design in the multivariate logistic regression models. However, we cannot evaluate whether the relevant time frame for the observed associations between air pollution reduction and improved respiratory health is long term (several years) or rather the year-to-year variability of air pollution levels, as recent Californian findings suggest (McConnell et al. 2003). For lifetime prevalence of asthma and hay fever, the relevance of the investigated change of exposure over a few years could be questioned, particularly for teenage children (eighth graders) who were exposed to higher air pollution levels in their early years of life, compared with first graders. Zmirou et al. (2004) report that exposure to traffic exhausts before the age of 3 years is associated with asthma in school children, but not lifelong exposures. In our data, no effect modification by age could be observed for asthma and hay fever, and their lifetime prevalence has been stable over the last decade in Swiss adolescents (Braun-Fahrländer et al. 2004).

We conclude that air pollution abatement measures implemented in Switzerland in the 1990s that resulted in moderately reduced air pollution exposures (SAEFL 2003; Kuebler et al. 2001) have successfully contributed to improved respiratory health in Swiss schoolchildren. Thus, not only dramatic changes (Heinrich 2003), but also modest improvements of ambient air pollution seem to be beneficial for children’s respiratory health. The larger reduction in symptom rates in areas with a stronger decrease in PM_10_ levels supports the causality of observed associations between air pollution and respiratory health in children. Our findings do not suggest a threshold for adverse effects of PM_10_, because we observed beneficial effects of rather small PM_10_ reductions in a moderately polluted environment. In urban regions and in the proximity of streets with high traffic volume, current PM_10_ levels still exceed limit values of the Swiss Clean Air Act (SAEFL 2003). Therefore, it can be assumed that there is still a potential for further improvement of both ambient air pollution and children’s health in Switzerland.

## Figures and Tables

**Table 1:** Number of participating children by health assessment phase and school grade.

	First phase	Second phase			
School grade [age (years)]	1992–1993^a^	1998–1999	1999–2000	2000–2001	Total 1992–2001
1st (6–7)	1,405	2,077	0	0	3,482
4th (9–11)	1,143	0	0	1,377	2,520
8th (13–14)	1,478	0	2,106	0	3,584
Total	4,026	2,077	2,106	1,377	9,591

a Surveys were conducted during a school year, which includes 2 calendar years.

**Table 2:** Definition of symptoms and diseases.

Symptom or disease	Positive answer to the following question(s):
Chronic cough	In the last 12 months, has your child had a cough associated with a respiratory infection lasting for more than 4 weeks?
Bronchitis	In the last 12 months, has your child had bronchitis?
Common cold	In the last 12 months, has your child had a common cold^a^?
Nocturnal dry cough	In the last 12 months, has your child had a dry cough at night, apart from a cough associated with a cold or a chest infection?
Conjunctivitis symptoms	In the last 12 months, has your child had itchy or irritated eyes when he/she did not have a problem with the nose? (not caused by chlorinated water)
Wheeze	In the last 12 months, has your child had wheezing or whistling in the chest?
Sneezing	In the last 12 months, has your child had a problem with sneezing, or a runny or blocked nose when he/she did not have a cold or the flu and this occurred during pollen season (March–September)?
Asthma	Has your child ever had asthma?
Hay fever	Has your child ever had hay fever?

a In the German translation (*grippe*), this includes the flu.

**Table 3:** Adjusted prevalence of health outcomes and their change across all, urban/suburban,^a^ and rural/alpine^b^ regions.

	Average of adjusted prevalence (%)^c^			
Symptom or disease	1992–1993 (95% CI)	1998–2001 (95% CI)	Absolute change	Percent change
Chronic cough				
All regions	12.0 (8.9–16.2)	7.9 (5.8–10.7)	−4.1	34.4
Urban/suburban	13.9 (10.6–18.2)	9.2 (6.9–12.2)	−4.7	34.1
Rural/alpine	8.2 (5.4–12.2)	5.3 (3.5–7.9)	−2.9	35.5
Bronchitis				
All regions	14.9 (11.2–19.6)	9.0 (6.7–12.0)	−5.9	39.9
Urban/suburban	15.3 (11.9–19.5)	9.2 (7.1–12.0)	−6.1	39.7
Rural/alpine	14.1 (9.9–19.9)	8.4 (5.8–12.1)	−5.7	40.3
Common cold				
All regions	35.0 (29.8–40.6)	26.1 (21.9–30.8)	−8.9	25.4
Urban/suburban	35.7 (30.9–40.7)	26.7 (22.8–30.9)	−9.0	25.2
Rural/alpine	33.7 (27.6–40.4)	25.0 (20.1–30.6)	−8.7	25.9
Nocturnal dry cough				
All regions	18.7 (14.6–23.7)	13.3 (10.3–17.0)	−5.4	29.0
Urban/suburban	20.7 (16.6–25.4)	14.8 (11.7–18.4)	−5.9	28.6
Rural/alpine	14.8 (10.7–20.2)	10.3 (7.5–14.3)	−4.5	30.2
Conjunctivitis symptoms				
All regions	19.7 (15.6–24.7)	15.2 (11.9–19.2)	−4.5	23.0
Urban/suburban	21.1 (17.1–25.7)	16.3 (13.1–20.1)	−4.8	22.7
Rural/alpine	17.1 (12.6–22.8)	13.0 (9.6–17.5)	−4.0	23.7
Wheeze				
All regions	8.2 (5.6–11.8)	6.1 (4.2–8.9)	−2.0	25.0
Urban/suburban	8.5 (6.1–11.9)	6.4 (4.6–9.0)	−2.1	24.9
Rural/alpine	7.4 (4.7–11.7)	5.5 (3.5–8.8)	−1.9	25.1
Sneeze				
All regions	8.9 (6.3–12.5)	7.2 (5.2–10.1)	−1.7	18.8
Urban/suburban	8.7 (6.4–11.7)	7.1 (5.2–9.5)	−1.6	18.8
Rural/alpine	9.3 (6.1–14.0)	7.6 (5.0–11.3)	−1.7	18.8
Asthma				
All regions	8.2 (5.7–11.8)	7.5 (5.2–10.6)	−0.7	8.7
Urban/suburban	7.5 (5.4–10.4)	6.8 (4.9–9.5)	−0.7	8.7
Rural/alpine	9.5 (6.2–14.4)	8.7 (5.8–13.0)	−0.8	8.5
Hay fever				
All regions	9.8 (7.1–13.5)	9.4 (6.9–12.7)	−0.4	4.6
Urban/suburban	9.4 (7.1–12.5)	9.0 (6.8–11.8)	−0.4	4.6
Rural/alpine	10.6 (7.2–15.5)	10.1 (7.0–14.6)	−0.5	4.5

CI, confidence interval.

a Urban/suburban regions: Anières, Bern, Biel, Geneva, Lugano, Zürich.

b Rural/alpine regions: Langnau, Payerne, Montana.

c Adjusted for age, sex, nationality, parental education, number of siblings, farming status, low birth weight, breast-feeding, child who smokes, family history of asthma, bronchitis, and/or atopy, mother who smokes, humidity, mode of heating and cooking, carpeting, pets allowed in bedroom, removal of carpet and/or pets for health reasons, person who completed questionnaire, month when the questionnaire was completed, number of days with the maximum temperature < 0°C, and belief of mother that there is an association between environmental exposures and children’s respiratory health.

**Table 4:** Distribution of covariates in the first and second health assessment phase (all regions combined).

Characteristic	1992–1993 (*n* = 3,024) *n* (%)	1998–2001 (*n* = 4,428) *n* (%)	*p*-Value^a^
Sex (male)	1,550 (51.3)	2,191 (49.5)	0.139
Nationality			
Swiss	2,288 (75.7)	3,214 (72.6)	0.003
Parental education^b^			
Low	446 (14.8)	500 (11.3)	< 0.0001
Low-middle	436 (14.4)	458 (10.3)	
Middle	949 (31.4)	1,294 (29.2)	
Middle-high	516 (17.1)	852 (19.2)	
High	677 (22.4)	1,324 (29.9)	
No. of siblings			
0	449 (14.9)	600 (13.6)	< 0.0001
1	1,729 (57.2)	2,341 (52.9)	
2	624 (20.6)	1,091 (24.6)	
≥3	222 (7.3)	396 (8.9)	
Farming^c^	117 (3.9)	183 (4.1)	0.57
Low birth weight (< 2,500 g)	340 (11.2)	547 (12.4)	0.146
Family history of disease^d^	1,490 (49.3)	2,418 (54.6)	< 0.0001
Breast-feeding (any)	2,436 (80.6)	3,829 (86.5)	< 0.0001
Mother smokes	800 (26.5)	1,102 (24.9)	0.127
Child smokes (8th graders; *n* = 2,661)	67 (6.4)	263 (16.3)	< 0.0001
Indoor humidity^e^	809 (26.8)	1,116 (25.2)	0.133
Central heating	243 (8.0)	520 (11.7)	< 0.0001
Cooking mode			
Electric	2,335 (77.2)	3,611 (81.6)	< 0.0001
Wood	71 (2.4)	85 (1.9)	
Gas	618 (20.4)	732 (16.5)	
Floor type			
Wood	545 (18.0)	1,798 (40.4)	< 0.0001
Single carpet	460 (15.2)	772 (17.4)	
Wall-to-wall carpet	2,019 (66.8)	1,867 (42.2)	
Pets			
No pets	1,451 (48.0)	2,031 (45.9)	< 0.0001
Pets in house	731 (24.2)	1,163 (26.3)	
Pets in bedroom	842 (27.8)	1,234 (27.9)	
Removal of carpet^f^	85 (2.8)	251 (5.7)	< 0.0001
Removal of pets^f^	68 (2.3)	96 (2.2)	0.816
Mother completed questionnaire	2,702 (89.4)	3,918 (88.5)	0.242
Environmental concern^g^	2,385 (78.9)	3,346 (75.6)	0.001
No. of cold days^h^			
gions	15	12	< 0.0001
Anières	10	3	< 0.0001
Bern	18	15	< 0.0001
Biel	13	8	< 0.0001
Geneva	10	5	< 0.0001
Langnau	20	19	0.02
Lugano	0	1	< 0.0001
Montana	21	38	< 0.0001
Payerne	22	16	< 0.0001
Zürich	21	17	< 0.0001

a Comparison of 1992–1993 and 1998–2001 using chi-square or *t*-tests as appropriate.

b Low: father and mother have no professional training; low-middle: father or mother has professional training of < 2 years; middle: father or mother has professional training of 2–4 years; middle-high: father or mother has academic training; high: father and mother have academic training.

c Family of child is full-time or part-time farming.

d Father and/or mother and/or siblings have asthma and/or atopy and/or chronic bronchitis.

e Mildew or water damage in the flat.

f Because of allergy or asthma of child.

g Mother believes that there is an association between environmental exposures and children’s respiratory health.

h Number of days with the maximum temperature < 0°C, assessed at the local fixed monitoring station.
